# Presacral Tumor: Insights From a Decade’s Experience of This Rare and Diverse Disease

**DOI:** 10.3389/fonc.2021.639028

**Published:** 2021-03-16

**Authors:** Zeyu Li, Min Lu

**Affiliations:** ^1^ Department of Colorectal Surgery, First Affiliated Hospital of China Medical University, Shenyang, China; ^2^ Department of General Surgery, Shengjing Hospital of China Medical University, Shenyang, China

**Keywords:** presacral tumor, retrospective cohort study, clinical presentation, diagnosis, surgical resection

## Abstract

**Background:**

Presacral tumors are a group of rare and heterogeneous tumors that arise from the potential presacral space between the rectum and sacrum. The low occurrence and diverse origins make the diagnosis and treatment of these tumors a challenge. The aim of the study was to retrospectively review patient demographics and to identify advantages and disadvantages in the diagnosis and treatment of these tumors.

**Methods:**

Retrospectively collected and reviewed data from patients who received treatment of presacral tumors at the First Affiliated Hospital of China Medical University between August 2009 and June 2019.

**Results:**

The data from forty-four patients (33 females) with a median age of 50 years who were diagnosed with a presacral/retrorectal tumor were analyzed. The majority of tumors were congenital (61.4%) and benign tumors are more common (59.1%). The median age of patients with benign tumor was significantly higher than that of malignant tumor. The most common symptoms were sacrococcygeal/perianal pain (56.8%) and mass (36.4%), and 8 out of 9 patients having lower limb symptoms diagnosed with malignant tumor. The tumor detection rate of digital rectal examination was 75% and more than 90% of all patients underwent one or more radiology imaging exams for tumor diagnosis. Every patient had a biopsy result. The most common type of tumor was presacral cyst (40.9%) with overall tumor median size of 5.6 cm. Thirty-one (70.5%) patients underwent surgery, most often *via* the posterior route (83.9%). Posterior route surgery had significantly shorter operation time and tumors operated *via* posterior route were significantly smaller. The survival rate after surgery was 100%. The median course of disease was 6 months and median follow-up was 25 months.

**Conclusions:**

Presacral tumors have low occurrence and are more frequently observed in females in their 30s and 50s indicating a possible link between tumor occurrence and hormonal changes. Patients with lower limb symptoms were more likely to have a malignant presacral tumor. Posterior route was the most commonly utilized surgical approach. Supplementary iodine tincture treatment of cysts ruptured in operation could potentially be helpful in reducing the chance of recurrence.

## Introduction

**Figure 1:**
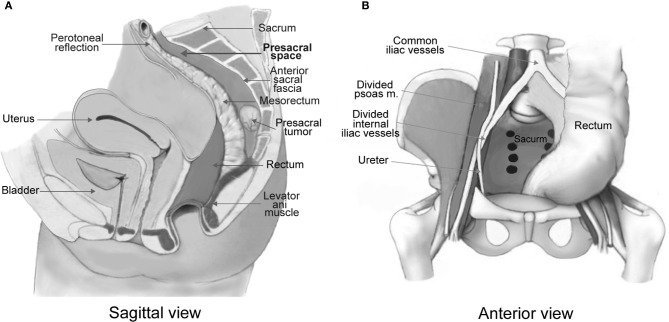
Anatomical illustration of a presacral tumor in relation with the presacral space **(A)**. The presacral space is formed with the rectum and mesorectum in the anterior, the anterior sacral fascia in the posterior, the levator ani muscle in the inferior, the peritoneal reflection in the superior **(A)**, and the iliac vessels and ureters laterally **(B)**. Illustrations are modified from Dozois and Marcos (2).

Presacral tumors refer to a group of rare and heterogeneous tumors that occur in the potential space between the rectum and the sacrum (1, 2) (**Figure 1**). Most of the published papers on these tumors are single case reports or studies on a limited number of cases. A few retrospective cohort studies were conducted mostly from large treatment centers with patients collected over a period of 1-5 decades (3–9). Tumor incidence was reported as 1.4–6.3 patients per year (1, 3–9). These heterogeneous tumors are difficult to classify because the tissues surrounding the presacral space are originated from the embryologic stem cells that later differentiated into three germinal layers which further develop into connective, osseous and neural tissues. The traditional Lovelady and Dockerty (10) classification and the more recent Uhlig and Johnson (11) classification system are currently utilized by surgeons.

Presacral tumors occur in both sexes at the median age of 45–50 years and more frequently in female (3, 12, 13). The fact that majority of patients are asymptomatic or show only non-specific symptoms such as pain in the perianal area or lower back, constipation or lower limb numbness (14–16) makes diagnosis a challenge. Misdiagnoses often happen and lead to delay of proper treatment and sometimes undesired consequences (1, 16).

Biopsy remains the gold standard for diagnosis of these tumors, but many advanced imaging modalities including MRI, CT scans have become as effective in making diagnosis and treatment plans. Complete surgical excision is still the best choice for these tumors because of the possibility of infection or the malignant tendency (5, 15).

The current study collected 44 cases of presacral tumors in one of the largest tertiary institutions in Liaoning province of China in the past decade. The aim of this study was to evaluate patient information from this institution and compare it to what has been published in other centers. The goal was to identify patient demographics as well as some of the advantages and disadvantages in diagnosing and treating presacral tumors.

## Materials and Methods

This study was conducted by retrospectively reviewing the surgical pathology and tumor registries at the First Affiliated Hospital of China Medical University. The research protocol was approved by the Medical Science Research Ethic Committee of the First Affiliated Hospital of China Medical University. Written informed consent was obtained from patients prior to their treatment. All patients who had a confirmed diagnosis of a benign or malignant presacral/retrorectal tumor between August 2009 and June 2019 were selected and all available data on each case were collected. Patients whose diagnose could not be confirmed by pathology or have no biopsy records were excluded from the study. In most cases, data collected include the demographics, clinical presentation, pre-surgery diagnosis, surgical approach, surgical margins, tumor pathology, adjuvant therapy, biopsy results, radiologic imaging, mortality, and local recurrence. We also conducted follow-up on available patients after patient identifications were uncovered.

Complications are reported based on the Clavien-Dindo classification (16). Literature review was conducted by searching English or Chinese peer reviewed articles including some of the Chinese articles with English abstracts.

### Statistical Analysis

Non-normally distributed quantitative variables were reported as median and range. Associations with quantitative variables between groups with different sample sizes were analyzed with the non-parametric Kruskal-Wallis one-way analysis of variance test by ranks, categorical variables were analyzed with Pearson’s Chi-Square test and survival differences were analyzed with Kaplan-Meier estimates. Statistical significance was defined as *p* < 0.05.

## Results

### General Patient Information

Forty-four patients were identified including 33 (75%) females and 11 (25%) males. The median age of all patients was 50 years (range 13–87 years) with the median age of female at 49 (range 13–87 years) and that of male at 55 (range 24–77 years). The average course of disease at presentation was 6 months (range 0.1–720 months). Twenty six patients were diagnosed with benign tumors whereas 18 were diagnosed with malignant tumors (**Table 1**). There were 31 patients who underwent surgery, eight patients received adjuvant therapy, and five patients were simply followed up and observed.

**Table 1:** Initial symptoms (patient may have more than one symptom).

Symptom	Benign		Malignant		No. of Patient
	Male	Female	Male	Female	
Sacrococcygeal/perianal pain	0	12	4	9	25 (56.8%)
Sacrococcygeal/perianal mass	2	10	2	2	16 (36.4%)
Bowel or urinary complaints	2	7	2	2	13 (29.5%)
Lower limb symptoms	1	0	3	5	9 (20.5%)
Physical exam or others	0	11	2	2	15 (34.1%)
Total Number	4	22	7	11	44

Patients with malignant tumors were significantly younger (*p* = 0.01214) with a median age of 38 years (range 13–87 years) while patients with benign tumors had a median age of 59 years (range 14–80 years).

**Figure 2:**
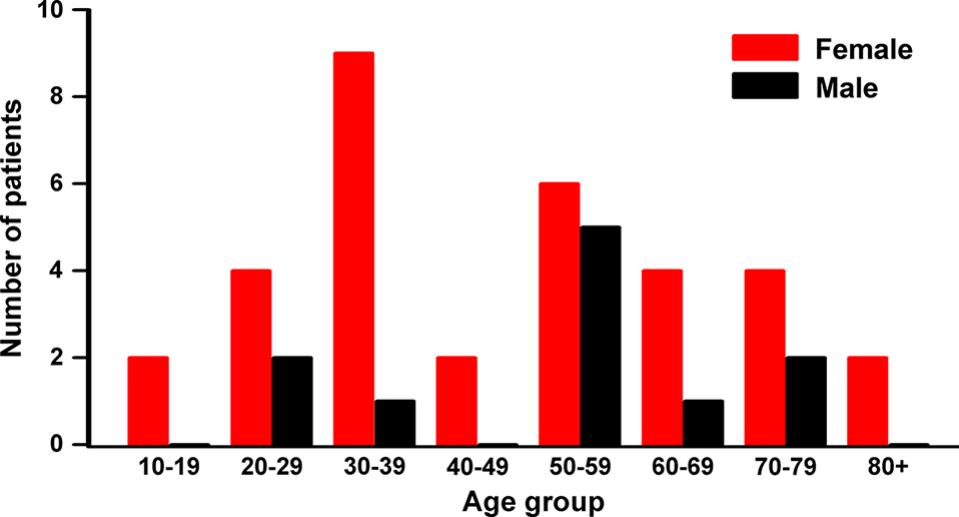
Patient distribution by gender and age groups. High occurrence age is the 50s in both male and female, while the highest in female is the 30s. Nonparametric Kruskal-Wallis ANOVA test showed significant differences between genders by age group (*p* = 0.0136), but not the median age between genders (p = 0.65452).

When patients were grouped according to age by decades, we found that the highest number of female patients is in the 30s (30–39 years; 9/33, 27.3%) followed by the 50s (6/33, 18.2%), while five out of 11 male patients are in their 50s (45.6%; **Figure 2**). Non-parametric Kruskal-Wallis analysis indicated that the number of female patients in each age group was significantly higher than the number of males (*p* = 0.01356), while there was no difference in the median age between genders *(p =* 0.65452).

The female patients in their 30s were mostly diagnosed with congenital tumors (8/9, 88.9%) which included six developmental cysts and two teratomas. The female patients in the 50s have a similar pattern with four congenital tumors (4/6, 66.7%) with three developmental cysts and one chordoma. Interestingly, these higher occurrences of the tumors in female patients in their 30s and 50s coincide with periods of dramatic changes in female hormones. Better understating of the contribution of female hormones to the development and growth of these congenital tumors could lead to novel and improved methods for the management of the presacral tumors in female patients.

### Clinical Presentations

The most common initial symptoms were sacrococcygeal and perianal pain (25, 56.8%) followed by sacrococcygeal and perianal mass (16, 36.4%) and dysfunction of bowel or urinary systems (13, 29.5%; **Table 1**). In addition, some lower limb symptoms (20.5%) such as numbness, radiating pain or movement problems have been reported. The overall tumor size of our cohort of patients is 5.6 cm (range 1.2–20 cm), the median tumor size for the surgical group is 5.6 cm (range 2.2–20 cm); that of the adjuvant therapy group is 5.36 cm (range 3.1–16.7 cm) while that of the untreated group is 6.4 cm (range 3.2–16 cm).

When compare presenting symptoms by malignancy, patients with benign tumors have pain less frequently (12/26, 46.2%) than the ones with malignant tumors (13/18, 72.2%), Pearson’s Chi-Square test showed a trend towards significance (*p* = 0.08609). It is worth noting that majority of patients (eight of nine cases, 88.9%) who had lower limb symptoms have malignant tumors (*p * = 0.00103, **Table 2**), and all female patients presenting these symptoms were diagnosed with malignant tumors (**Table 1**). There was no difference in tumor sizes between benign and malignant cases (*p* = 0.51606, **Table 2**).

**Table 2:** Comparison between patients with benign and malignant tumors.

Tumor type	Benign	Malignant	*p* Value		
Age (year)	59 (14–80)	38 (13–87)	**0.01214**		
Tumor size (cm)	4.805 (1.2–20)	6.3 (3.1–20)	0.51606		
Follow-up (mon)	27 (3–69)	25 (6–93)	0.91708		
Surgical case	23 (88.5%)	8 (44.4%)	**0.00114**		
Sacrococcygeal/perianal pain	12 (46.2%)	13 (72.2%)	0.08609		
Sacrococcygeal/perianal mass	12 (46.2%)	4 (22.2%)	0.10470		
Bowel or urinary complains	9 (34.6%)	4 (22.2%)	0.37568		
Lower limb symptoms	1 (3.8%)	8 (44.4%)	**0.00103**		
Recurrence rate*	23.5%(4/17)	71.4%(5/7)	**0.02746**		
Total cases	26	18	

*Follow-up patients; bold numbers represent p < 0.05.

### Diagnostic Modalities and Tumor Characteristics

**Figure 3:**
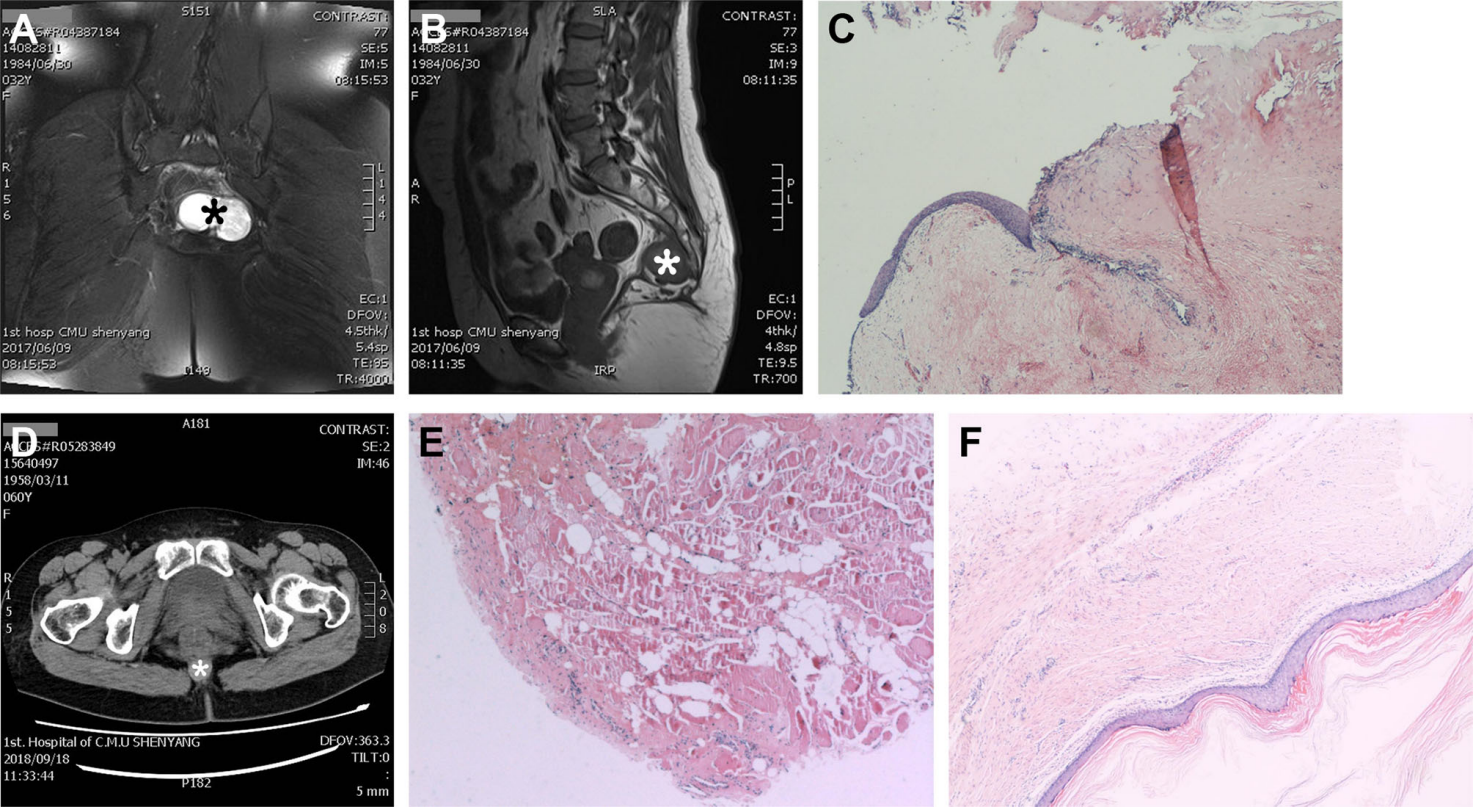
Representative cases of the use of diagnostic imaging and pathology. MRI pelvis with axial view **(A)** showing a 6.2 cm × 4.4 cm × 4.1 cm tumor (*), and sagittal view **(B)** showing the relationship between tumor (*) and the walls of the presacral space. **(C)** Biopsy showing that the tumor contains variety of tissues and surrounded by a cystic wall, which is consistent with a presacral teratoma **(A–C)**. **(D)** CT scan image showing a size 2.0 cm × 2.0 cm round tumor (*) with smooth edge and biopsy **(E)** confirms that it is a benign epidermoid presacral tumor. **(F)** Patient who was diagnosed with a perianal tumor on digital rectal examination and confirmed with biopsy, the Hematoxylin-Eosin stain of the cyst wall and content indicating a presacral cyst.

There were 20 patients who had digital rectal examination (DRE), 15 (75%) of which had positive tumor palpation. There were 37 (84.1%) patients who had preoperative or postoperative biopsies in our hospital to confirm the tumor pathology, and seven (15.9%) were confirmed by other institutions. Most patients (41, 93.2%) had preoperative diagnostic imaging of MRI/CT/B-mode ultrasound/PET-CT (one or multiple), and the other three (6.8%) were diagnosed by DRE combined with intra-operative findings (examples shown in **Figure 3**). Among the 41 patients with diagnostic imaging, CT scan along was performed in 20 (58.5%), MRI along in 12 (46.3%), and ultrasound along in two (4.9%) patients; a combination of CT and MRI in four (9.6%), MRI and ultrasound in three (7.3%) patients. We found that the accuracy of the MRI is at 89.5%, a little bit higher than the CT scan (83.3%), though not statistically significant.

In our cohort of patients, the most common presacral tumors were congenital ones (28, 63.6%) including developmental cysts (18, 40.9%) with 14 (77.8%) tailgut cysts, two epidermoid and two dermoid cysts followed by chordomas (6, 13.6%) and teratomas (4, 9.1%; **Table 3**). Majority of the tumors were benign (26, 59.1%); among the 18 cases of malignant tumors (40.9%), 13 were originated in the presacral space which included six chordomas and two teratomas, and the other five were metastasized ones (**Table 3**).

**Table 3:** Tumor classification according to Uhlig and Johnson system (3).

Types of presacral tumor	N = 17
Congenital (28)	
Developmental (18)	
Epidermoid	2
Dermoid	2
Tailgut cyst	14
Teratomas (2*)	4
Chordomas*	6
Inflammatory: abscesses	1
Neurogenic	
Chordal meninginoma*	1
Miscellaneous (14)	
Plasmocytoma*	2
Mucinous adenocarcinoma*	1
Epithelial neoplasms	1
Smooth muscle tumor	1
Giant cell tumor of sacrum	1
Clear cell sarcoma of soft tissue*	1
Retrorectal benign nodule	1
Vascular tumor with abscess	1
Secondary malignant tumor**	1
Metastatic adenocarcinoma**	4

*Original malignant tumor. **metastatic tumor.

### Clinical Managements

Thirty-one patients (70.5%) received surgery, among which two had previously received rectal cyst excision and perianal abscess removal from other institutions. The 13 non-surgical patients included eight patients with malignant tumors, four of whom (9.1%) received adjuvant therapy (previously underwent surgery), and four (9.1%) received radiation/chemotherapy only; and the presacral tumors of the other five patients (11.4%) were not treated by patients’ requests, four of which discovered the presacral tumors incidentally when treating the original diseases.

Complete tumor resection was the goal of the presacral tumor operation. Among the operations, 21/31 (67.7%) were surgical resections alone and 10/31 (32.3%) were presacral cysts resections combined with supplementary iodine tincture cauterization that resulted in similar overall cure rate (87.5% *vs.* 88.9%, respectively). There were more patients with benign tumor who underwent surgeries (23/26, 88.5%) than the ones with malignant tumor (8/18, 44.4%; *p* = 0.00114; **Table 2**).

The most common surgical approach was the posterior approach *via* sacrococcyx or perineal (26/31, 83.9%), which had the shortest median operation time of 45 min (range 20–315 min; *p* = 0.00702) when compared with an anterior approach *via* abdomen (3/31, 9.7%) and a combined anteroposterior approach for those with larger tumors (2/31, 6.4%; **Table 4**). The median tumor size of the posterior surgery group was 5.3 cm (range 2.2–9.1 cm) which was also significantly smaller than the other two groups (*p* = 0.03479; **Table 4**). There was no difference in tumor size between male and female patients who underwent surgery (*p* = 0.57217).

**Table 4:** Comparison of three surgical approaches.

Surgical approach	Posterior	Anterior	Combined	Total	*p* Value
No. of Patient	26	3	2	31	
Course of disease (mon)	12 (0.1–720)	24 (8–240)	19 (2–36)	6 (0.1–720)	0.46199
Operation time (min)	45 (20–315)	195 (120–210)	429.5 (420–439)	60 (20–439)	**0.00702**
Hospital stay (day)	7 (2–177)	12 (9–19)	14.5 (14–15)	8.5 (2–177)	0.31613
Tumor size (cm)	5.3 (2.2–9.1)	7.6 (7.5–20)	12.85 (5.7–20)	5.6 (2.2–20)	**0.03479**
Follow-up (mon)	30 (3–93)	20.5 (16–25)	17 (17–17)	25 (3–93)	0.51029
Recurrence rate*	8 (21^*^;38.1%)	0 (2^*^;0%)	1 (1^*^;100%)	9 (24^*^;37.5%)	0.25281
Survival rate*	100%	100%	100%	100%	0.99999

*Follow-up patients only; bold numbers represent p < 0.05.

### Follow-Up and Complications

There were 34 follow-up consultations conducted (77.3%) and the median follow-up time was 25 months (range 3–93 months). Among the 24 follow-ups of the 31 patients in the surgery group, nine recurred (37.5%) and all survived (**Table 4**). Majority of the recurred patients (6/9, 66.7%) underwent a second surgery, and only one recurred after two operations. The remaining patients (3/9, 33.3%) chose non-operational treatment because they were asymptomatic. Of the 10 follow-up patients in the non-surgical groups, two patients were in the radiation/chemotherapy group one of which recurred with no symptom and the other one died after 18 months from the malignant tumor; four follow-up patients were in the post-operative radiation/chemotherapy group including one recurrence, two deaths from the malignant tumors after four and 66 months of survival (respectively), and one remained in treatment; the other four were untreated patients of which three died with one due to the presacral malignant tumor and two from other pre-existing diseases.

**Figure 4:**
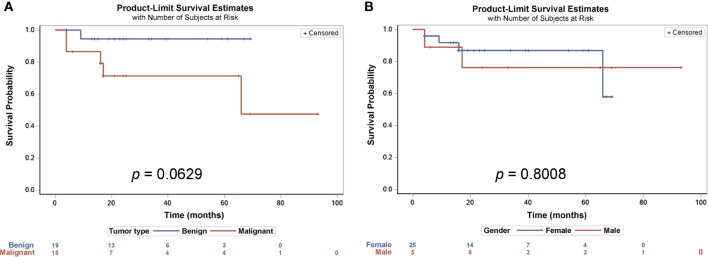
Survival probability estimates of patients with presacral tumors. Kaplan-Meier estimates analysis showed that there was a trend for patients with benign tumors having a longer survival rate, though not statistically significant **(A)**, but there were no differences between male and female patients **(B)**.

There were no significant differences between the three surgical approaches in recurrence rate (*p* = 0.25281) and survival rate (**Table 4**). When divided patients with tumor malignancy, malignant tumors have significantly higher recurrence rate (*p* = 0.02746, **Table 2**), and there was a trend that patients with benign tumor have higher survival rate than the ones with malignant tumor (*p* = 0.0629; **Figure 4A**), but there was no gender difference in overall survival (**Figure 4B**).

Seven patients developed postoperative complications, five of which had poorly healed incisions (Grade I) after a posterior surgical approach, one had rectal fistula (Grade IIIa) following an anterior approach and another one who underwent a combined approach surgery plus coccygectomy had lower limb movement dysfunctions (Grade Id).

## Discussion

Cases of presacral tumors diagnosed and treated at a large regional hospital for the past 10 years were reviewed to gain insights in the occurrence rate, diagnosis, and treatment of this rare tumor. The data indicate that these tumors are most commonly seen in female patients in their 3^rd^ and 5^th^ decade of age and that the majority of these tumors can be characterized as congenital presacral tumors. The high incidence coincides with the ages when female hormonal changes are the most pronounced. Lower limb symptoms were predictive of malignant tumors and we reported for the first time that surgical resection of presacral cysts supplemented with iodine tincture treatment could have helped to reduce the risk of recurrence.

Presacral tumors, also referred as retrorectal tumors, are very rare with reported cases in the literature of only 1.4–6.3/year (1, 3–9). The occurrence in this patient cohort was 4.4 cases/year, and the number of female patients is significantly higher than that of male patients in every age group especially in the 3^rd^ and 5^th^ decades. Female fertility starts decreasing significantly in the third decade (17) and menopause happens around the beginning of the fifth decade of age (18). It is notable from our data pool that females in the 30s and 50s age groups had the highest incidence of these tumors coinciding with the periods of drastic hormonal fluctuations. Due to the rarity and diversity of these tumors, this phenomenon has apparently not been documented before. In support of our findings, the Buchs et al. (4) study of 16 cases included 13 female patients, among which the highest numbers were in their 30s (6, 46.2%) and 50s (3, 23.1%). Other evidence shows that a presacral benign cyst had strong estrogen receptor immunohistochemical staining in both the cyst-lining cells and the tumor cells in humans (19) and that age-related difference of teratoma growth rate in female mice was due to the changes in the levels of the estrogen and progesterone (20). Based on the evidence above, we postulate that dramatic changes in female hormones may contribute to the development of congenital presacral tumors. This may represent a potential avenue for discovering new treatment strategies. For example, when a female patient presenting non-specific sacral/anal pain that is in her 30s or 50s, a presacral congenital tumor should be considered; estrogen or progesterone receptor inhibitors may be of benefit to prevent tumor growth providing a potential non-invasive treatment option for these tumors especially for patients who are asymptomatic. Such endocrine inhibitors have been the primary systemic treatment for estrogen or progesterone receptor positive breast cancer (21).

It is not unusual for patients with presacral tumor to be asymptomatic. Most of the initial symptoms are related to tumor compression or invasion of the surrounding tissues and organs (22). In our study, five patients (11.4%) showed no initial symptoms as similarly reported in some studies (12, 23, 24), while other studies showed a higher percentage of patients with no symptoms (up to 50%) (8). Besides the pain and mass found in majority of patients, we found 8 out of 9 patients who have lower limb symptoms are diagnosed with malignant tumors. Evidence indicates that the lower limb symptoms such as lumber radiculopathy were caused by malignant tumors invading the spine (25), and it is important when presenting with lower limb symptoms patients should be carefully examined for evidence of a malignant presacral tumor.

The frequency of malignant tumors in our cohort of patients is 40.9% which is similar to previous studies (3, 26). The malignant tumor develops differently between genders, and our cohort has higher frequency in males (1) while others reported more in females (3). When compared to the benign tumors, the median age of our patients with malignant tumors is much younger (*p* = 0.01214), whereas the recurrence rate of the malignant tumor is much higher (*p* = 0.02746, **Table 2**). The survival rate between benign and malignant tumors was trending towards being statistically different (*p* = 0.0629, **Figure 4A**), but may not have reached significance levels on the account of the fact that many of the patients had the low-grade malignant tumor chrodoma which is believed to have longer survival rate after tumor resection (27). In addition, the follow-up period may not have been long enough for the differences to be manifested. The fact that the median age of the patients with a malignant tumor in the present study is much younger and the recurrence rate is much higher underscores the need for early detection and early treatment of any presacral tumors despite the malignancy.

Surgery is and has been the pillar of the presacral tumor management. Ideally, surgery should remove the entire presacral tumor. In our institution, the posterior approach usually was performed using transsacrococcygeal or transperineal technique. Most (83.9%) of the tumors in our study were removed through the less invasive posterior approach which had the shortest operation time and short hospital stays (**Table 4**) in addition to an overall post-operational survival of 100%. It is also worth noting that in our study, 10 of the 18 patients with cystic presacral tumor received surgical resection followed by iodine tincture cauterization treatment. Iodine tincture is an alcohol-based solution used as an antiseptic and disinfectant. The iodine in the solution causes protein denaturation and necrosis of the cyst wall (28). Our study was the first to report the use of iodine tincture for treatment of presacral cysts even though it has been used for treating other types of cyst (29–32). This procedure was used in patients with cysts that had intra-operation capsule ruptures or when the cyst decompression was necessary during surgery. This is especially of consequence since capsule rupture is a common occurrence during surgical resection of presacral cysts (33, 34). The use of 2% iodine tincture solution to rinse the anterior sacral space can prevent seeding caused by residue cystic contents and in turn reduce the chance for tumor to recur. As a result, the overall cure rate with the supplementary iodine tincture treatment has reached similar level to that of the surgical resection alone (88.9% *vs.* 87.5%, respectively). A limited number of patients and follow-up in our study suggest a need for more research regarding the use of iodine tincture solution.

It is difficult to identify critical factors in retrospective study of a rare disease. While previous studies on presacral tumors were similarly based on a relatively small number of patients with median follow-up periods ranging from several months to 2 years (3–5, 9, 35), the present study is able to report new findings related to this rarely encountered tumor. In summary, presacral tumors are more common in females of 30 and 50 years of age when dramatic hormonal changes occur. Patients are often asymptomatic with palpable mass. Patients with lower limb symptoms are highly suspicious of a malignant presacral tumor. Posterior route is the most utilized approach because of the shorter operation time and hospital stays. Supplementary iodine tincture treatment could be of help in preventing cyst content seeding especially when the cyst was ruptured during surgery, which in turn lower the chance of these tumors to recur.

## Data Availability Statement

The original contributions presented in the study are included in the article/supplementary material. Further inquiries can be directed to the corresponding author.

## Ethics Statement

The studies involving human participants were reviewed and approved by the Medical Science Research Ethic Committee of the First Affiliated Hospital of China Medical University. Written informed consent to participate in this study was provided by the participants’ legal guardian/next of kin.

## Author Contributions

ZL conceived and designed the study, collected patients’ data, analyzed the data, and wrote the manuscript. ML provided critical comments for the manuscript. All authors contributed to the article and approved the submitted version.

## Funding

This study is supported by Liaoning Province Natural Science Foundation grant No.2019-ZD-0744 to ML.

## Conflict of Interest

The authors declare that the research was conducted in the absence of any commercial or financial relationships that could be construed as a potential conflict of interest.
